# Potentiation of antitumour activity of docetaxel by combination with trastuzumab in a human prostate cancer xenograft model and underlying mechanisms

**DOI:** 10.1038/sj.bjc.6603553

**Published:** 2007-01-09

**Authors:** M-E Legrier, S Oudard, J-G Judde, C Guyader, G de Pinieux, K Boyé, P de Cremoux, B Dutrillaux, M-F Poupon

**Affiliations:** 1 Section Recherche, Institut Curie, U612 INSERM, 26 rue d'Ulm, Paris 75248, France; 2 Cancérologie Médicale, Hôpital Européen Georges Pompidou, 20 rue Leblanc, Paris 75015, France; 3 Anatomie Pathologique, Hôpital Cochin, Paris 75005, France; 4 Section Médicale, Institut Curie, Paris 75248, France

**Keywords:** prostate adenocarcinoma, drug evaluation, potentiation, protein expression

## Abstract

Antitumour activity of docetaxel (Taxotere®) in hormone-dependent (HD) and hormone-independent (HID) prostate cancer PAC120 xenograft model was previously reported, and its level was associated with HER2 protein expression. In the present study, we evaluate the antitumour effects of docetaxel combined with trastuzumab (Herceptin®), an anti-HER2 antibody. Although trastuzumab alone had no effect on tumour growth, it potentiated the antitumour activity of docetaxel in HD tumours and more strongly in HID variants. Using the HID28 variant, we show that docetaxel treatment of tumour-bearing mice induces an increased HER2 mRNA expression of the tyrosine kinase receptor of 25-fold 24 h after docetaxel treatment, while HER2 protein and p-AKT decreased. This was followed by an increase of HER2 protein 3 days (two-fold) after docetaxel treatment and by a strong HER2 release in the serum of treated mice; expression of phospho-ERK, p27, BCL2 and HSP70 concomitantly increased. Similar molecular alterations were induced by docetaxel plus trastuzumab combination, except for that there was a transient and complete disappearance of AR and HSP90 proteins 24 h after treatment. We show that in addition to its known effects on tubulin and mitotic spindles, docetaxel induces complex signalisation pathway mechanisms in surviving cells, including HER2, which can be pharmacologically targeted. This study suggests that the docetaxel/trastuzumab combination may prove an effective therapeutic approach for HER2-expressing hormone-refractory prostate cancer.

Escape from androgen ablation therapy is a constant feature of prostate cancer progression, leaving patients with few therapeutic options. Hormone-refractory prostate cancer (HRPC) was regarded as chemoresistant, until the recent demonstration of taxane activity (Petrylak *et al*, 1999). A preclinical *in vivo* study using our PAC120 model of hormone-dependent (HD) human prostate cancer xenograft confirmed these data (de Pinieux *et al*, 2001; Oudard *et al*, 2003). A randomised phase II study demonstrated the efficacy of docetaxel-based chemotherapy vs a mitoxantrone–prednisone combination, significantly decrease PSA and prolongs time to progression of metastatic HRPC patients (Oudard *et al*, 2005). Despite these encouraging preclinical and clinical data, the therapeutic efficacy of taxane-based chemotherapy of HRPC remains modest. A better knowledge of the mechanisms of tumour hormonal escape and of key molecular targets is needed to design more effective combination therapies for HRPC.

Several preclinical studies have implicated the ERBB family of tyrosine kinase receptors, especially HER2, in the progression of prostate cancer to a hormone-refractory state. The CWR22 hormone-independent prostate cancer xenograft expresses HER2 and its growth is inhibited *in vivo* by an anti-HER2 antibody (Agus *et al*, 1999; Mendoza *et al*, 2002). Increased hormone independence in prostate cancer cells was associated with androgen receptor (AR) phosphorylation mediated by transfected HER2 (Craft *et al*, 1999; Yeh *et al*, 1999). A recent study in prostate cancer cell lines showed that an HER2/ERBB3 signalling pathway protected the AR from degradation by the ubiquitin–proteasome pathway and enhanced AR transcriptional activity at low androgen concentrations (Mellinghoff *et al*, 2004). Studies of clinical prostate cancer samples, however, have produced mixed data, failing to firmly establish a relationship between HER2 overexpression and progression to hormone independence (Osman *et al*, 2001; Shi *et al*, 2001; Savinainen *et al*, 2002; Calvo *et al*, 2003). HER2 gene is overexpressed in 20–25% of invasive breast cancers and in 60% of HRPC. Trastuzumab has antitumour activity in patients with HER2-overexpressing breast cancer. A phase II clinical trial of trastuzumab and docetaxel with HER2-overexpressing prostate cancer has recently been conducted (Lara *et al*, 2002) and needs to be completed.

In this study, we report the improvement of the docetaxel response of prostate cancer xenografts PAC120 by combination with trastuzumab (Herceptin®, Roche, Neuilly sur Seine, France), a humanised anti-HER2 antibody, and the idea is to understand tumour biological mechanism after combination. We show that docetaxel induces an increase of tumour HER2 expression. Trastuzumab, an anti-HER2 antibody which binds the ectodomain of the HER2 receptor, markedly potentiates the antitumour activity of docetaxel, an effect that is associated with early complete degradation of AR and HSP90 proteins. This potentiation was more pronounced in terms of survival delay and complete tumour regression in hormone-independent (HID) tumour variants of PAC120. The data suggest that docetaxel triggers an HER2-mediated signalling pathway, which protects tumour cells from taxane-induced alterations. The induction of HER2 opens a way for the design of effective combination therapy in prostate cancer.

## MATERIALS AND METHODS

### *In vivo* studies

PAC120 was established as xenograft by serial subcutaneous grafting of prostate cancer tissue into 8-10-week-old Swiss nu/nu male mice (bred and maintained at the Institut Curie SPF animal facility), as published (de Pinieux *et al*, 2001). The original tumour xenotransplant was obtained by transurethral resection of a locally recurrent primary prostate adenocarcinoma. PAC120 has a Gleason grade 9 (5+4) and is HD. Hormone-independent variants were derived from PAC120-bearing mice after bilateral orchidectomy. After tumour regression and regrowth, the tumours were maintained under androgen deprivation by transplantation into castrated males. Two HID variants, designated HID25 and HID28, were selected and used after the fourth passage. Experiments were performed under supervision of Ile de France-I ethical committee, according to French regulations.

Tumour fragments (30 mm^3^) were grafted in the interscapular subcutaneous tissue, one fragment per mouse, under anaesthesia (Oudard *et al*, 2003). Tumour take was almost 100%. Tumour-bearing mice were randomly distributed into groups of 7–12 assigned to control or treatment. Treatment was administered when the tumours reached 60–200 mm^3^ in size. Docetaxel (Taxotere®, Aventis Laboratories, France) was extemporaneously prepared according to the procedure used for the clinical compound, and injected intraperitoneally (i.p.) at a dose of 20 mg kg^−1^ every 3 weeks, as already published (Oudard *et al*, 2003). Trastuzumab (Herceptin®, Roche, France) was administered weekly by the i.p. route at 10 mg kg^−1^. The combination treatment was concurrently given on day 1 and every 3 weeks, with weekly administration of trastuzumab. These experiments were repeated twice for HID-25 and three times for PAC120 and HID28. For histological and gene/protein expression analyses, mice bearing PAC120 and HID tumours were killed at different times after treatment, as indicated in the results section.

Tumour growth was assessed by measuring two perpendicular diameters with calipers, twice weekly. Tumour volume (*V*) was calculated by *V*=*a*^2^ × *b*/2, where *a* is the width in mm and *b* is the length in mm. Individual tumour volume relative to initial size (*RTV*) and mean relative tumour volume SD per group were calculated. Tumour growth inhibition (TGI) was calculated as the ratio of RTV in the treated to control group at a given time × 100. Tumour growth delay (TGD) was the time in days required for tumours to achieve a five-fold increase in initial volume. Median survival was the time in days corresponding to the death of 50% of mice per group, mice being ethically killed when their tumours reached a volume of 2000 mm^3^. Statistical significance of the differences was calculated using the two-sided Student's *t*-test.

### Histology and immunohistochemical studies

PAC120, HID25 and HID28 tumour samples were removed from mice and immediately fixed in 10% formalin solution and embedded in paraffin. The mitotic index was determined as the average mitotic count in 10 high-power fields from analysis of HES (haematoxylin and eosin safran)-stained 4-*μ*m-thick sections. The apoptotic index was determined as the percentage of apoptotic cells stained by *asp*175-cleaved anti-caspase 3 antibody (Cell Signaling, Beverly, MA, USA), in 300 counted cells.

Immunostaining was performed on 4 *μ*m paraffin-embedded sections. Endogenous peroxidase activity was blocked by incubation of samples in 0.3% hydrogen peroxide in methanol for 30 min. Tissue slides were microwaved in 0.01 M sodium citrate buffer (pH 6) near boiling for 20 min. The sections were incubated with a blocking solution (5% horse serum, 1% bovine serum albumin, 0.1% sodium azide, in PBS) for 30 min. Primary antibodies for HER2/neu/erbB2 (Zymed Laboratories, San Francisco, CA, USA) and HSP90 (Calbiochem, Fontenay-sous-Bois, France) were incubated at room temperature for 1 h or at 4°C overnight, at 1 : 50 dilution. After incubation with a biotinylated secondary antibody (RTU Vectastain Elite kit, Vector Laboratories, Burlingame, CA, USA), the immunohistochemical reaction was visualised using the avidin–biotin–peroxidase complex (ABC Vectastain Elite kit, Vector Laboratories, Burlingame, CA, USA) with diaminobenzidine tetrahydrochloride (DAB). Sections were counterstained by Mayer's haematein solution. For each antibody, appropriate positive and negative controls were tested simultaneously.

### Western blotting and ELISA protein analyses

Tumour samples (three tumours per treatment group) were homogenised in NP40 lysis buffer (containing 0.5% Nonidet P40 in 50 mM Tris–HCl, pH 8, 120 mM NaCl, 1 mM EDTA, 5 mM NaF, 1 mM Na_3_VO_4_ and protease inhibitor cocktail tablets (Roche Applied Science, France)). Tumour lysates were incubated for 1 h at 4°C with constant stirring. Protein samples (15–20 *μ*g protein) were run on SDS polyacrylamide mini-gels and transferred to PVDF membranes. Blots were incubated overnight at 4°C with the diluted primary antibody. After 1 h of incubation at room temperature with the appropriate peroxidase-linked secondary antibody, immunoreactivity was detected by chemoluminescence using an ECL kit (Amersham, Buckinghamshire, UK). Antibodies were used at 1 : 50–1 : 2000 dilution. Total beta-tubulin (Sigma Aldrich, Saint-Quentin, France); HER2/neu/erbB2 (Zymed Laboratories); phospho-p44/42 MAPK (ERK1/2, Thr202/Tyr204), phospho-AKT (Ser 473) (Cell Signaling Technologies, Beverly, MA, USA); p27/KIP1 (Oncogene Research Products, Fontenay-sous-Bois, France); BCL-2 (DAKO, Danemark) Androgen receptor (Upstate Biotechnology, Euromedex, Souffelweyersheim, France); HSP90, (Calbiochem, Fontenay-sous-Bois, France), HSP70 (Interchim, Montluçon, France); Actin (Santa Cruz Biotechnology Inc., Santa Cruz, CA, USA). Relevant HPR-conjugated goat or rabbit antibodies (Santa Cruz Biotechnology) were used for detection of primary antibody. For quantitative analysis, the intensity of the signals was determined using ImageJ software then normalised relative to actin and control group.

For the measurement of serum HER2 levels, mice (three per group) were treated and bled 3 days after docetaxel treatment and the serum harvested and frozen at −20°C until use. Three mice were used as controls. The seric HER2 levels were measured by an ELISA kit at a densitometry of 492 nm (HER-2/neu Microtiter ELISA, Dako Cytomation, Carpinteria, CA, USA) according to the manufacturer's protocol.

### Real-time reverse transcription (RT)–polymerase chain reaction (PCR) analysis of gene expression

Tumour samples (five tumours from each treatment group) were harvested and snap frozen in liquid nitrogen. Total RNA was prepared with Trizol as described by the manufacturer (Invitrogen, Gercy Pontoise, France). RNA quality was confirmed by the RNA 6000 assay (Agilent Technologies 2100 Bioanalyser, Massy, France). Reverse transcription of RNA was performed by random priming (cDNA cycle Kit, Invitrogen) using 2 *μ*g of total RNA. Real-time RT–PCR and the design of primers were as described (Legrier *et al*, 2004). Primers designed with the Primer Express software (ABI, Les Ulis, France) were the following: *AR*: S: CTTGTGTCAAAAGCGAAATGGGC, AS: CAAAACATGGTCCCTGGCAGTC; *HER2*: S: TGGAGACCCGCTGAACAATAC, AS: CCTTTCAAGATCTCTGTGAGGCTT; *HPRT*: S: GCTTTCCTTGGTCAGGCAGTATAA, AS: AAGGGCATATCCTACAACAAACTT. For normalisation, transcripts of the *HPRT* gene encoding human hypoxanthine phosphoribosyltransferase, previously identified as the most stable reference gene for this experimental sample set using the geNorm software (Vandesompele *et al*, 2002), was used as endogenous RNA controls. Data represented median of five tumours for each treatment group and were expressed relatively to control group. Each PCR reaction was performed in triplicate.

## RESULTS

### Response of PAC120 and HID variants xenografts to docetaxel alone or combined with trastuzumab

**Figure 1:**
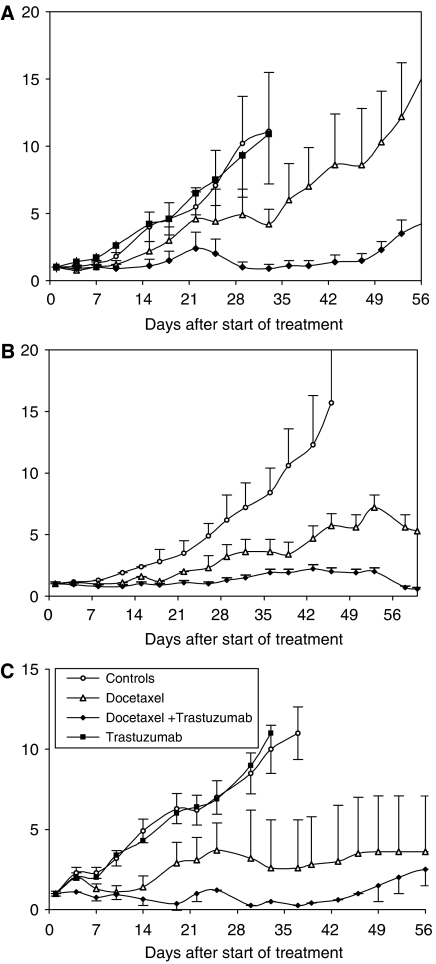
Effect of docetaxel and trastuzumab, alone or combined, on the growth of PAC120, HID25 and HID28 prostate cancer xenografts. Tumour growth curves in mice that were untreated (empty circles), treated with docetaxel, 20 mg kg^−1^ every 3 weeks (empty triangles), trastuzumab, 10 mg kg^−1^ weekly (filled squares) and docetaxel plus trastuzumab (filled lozenges). (**A**) Growth curves of the PAC120 HID prostate cancer xenograft. (**B**) Growth curves of the HID25 HID prostate cancer xenograft grown in castrated mice. (**C**) Growth curves of the HID28 HID prostate cancer xenograft grown in castrated mice. Bars represent s. d.

Following transplantation, PAC120 xenografts required 40 days on average to reach a volume of 100 mm^3^, with a tumour take of almost 100%. Docetaxel treatment was well tolerated, without significant loss of weight. Docetaxel inhibited tumour growth by 63% at day 33 (*P*=0.13) and prolonged the TGD 1.8-fold (Figure 1A; Table 1). One complete tumour regression was registered. Trastuzumab alone had no effect on the tumour, and was perfectly tolerated. When given with docetaxel, trastuzumab potentiated its antitumour effect, leading to a tumour growth inhibition of 85% at day 33 (*P*<0.03). Transient tumour regressions were observed in seven out of eight, one leading to a complete regression. The difference in TGI between the docetaxel plus trastuzumab combined treatment and the treatment by docetaxel alone was statistically significant (*P*=0.05 at day 51). When docetaxel was split into two doses of 10 mg kg^−1^, given at days 1 and 8 every 3 weeks, no reduction of tumour growth was obtained and the combination with trastuzumab did not induce any response (data not shown).

The HID28 and HID25 xenografts growing in castrated males reached a 100 mm^3^ volume after 55 and 60 days, respectively, with a tumour take of almost 100%. Docetaxel inhibited the growth of HID25 by 64% at day 45 (*P*=0.02) and prolonged the TGD 2.3-fold (Figure 1B, Table 1). The treatment was maintained for 120 days (six cycles of treatment) without any tumour growth (data not shown) and was well tolerated (no loss of weight). The trastuzumab plus docetaxel combination treatment reduced tumour size by 87% at day 45 (*P*<0.001), leading to complete tumour regressions in six out of mice at day 180. Docetaxel inhibited the growth of HID28 by 77% at day 38 (*P*<0.01), prolonged the TGD by 5.4 times (Figure 1C, Table 1) and induced complete tumour regressions in five out of eight mice. Combination of trastuzumab with docetaxel potentiated tumour growth inhibition (98% at day 38) and prolonged the survival up to 132 days, TGD being not reached. Trastuzumab alone had no effect on tumour growth (as shown in HID28, Figure 1C).

### Alteration of tumour histology after docetaxel or docetaxel/trastuzumab combination treatment

**Figure 2:**
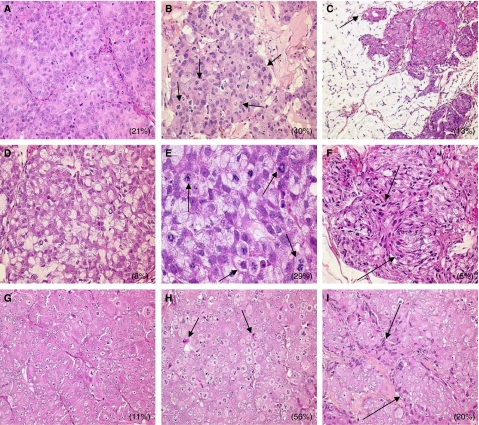
Histological features of PAC120, HID25 and HID28 xenografts (HES staining, × 200 magnification). Left panels, untreated tumours; middle panels, docetaxel-treated tumours; right panels, docetaxel plus trastuzumab-treated tumours. (**A**) PAC120 tumours were characterised by a Gleason score of 9 (5+4) with compact sheets of tumour cells surrounded by a fine murine stroma. (**B**) At 3 days after docetaxel treatment, accumulation of mitotic cells is visible (arrows). (**C**) At 47 days after treatment with docetaxel plus trastuzumab, the tumour has markedly regressed, leaving large areas of empty fibro-adenomatous tumour stroma (arrow), invaded by murine cells. (**D**) HID25 tumours display mucinous differentiation with principally signet-ring cells. (**E**) At 3 days after docetaxel treatment, accumulation of numerous and abnormal mitotic cells (arrows) (HES × 400). (**F**) At 3 days after treatment with the docetaxel plus trastuzumab combination, presence of elongated tumour cells with hyperchromatic nuclei (arrows). (**G**) HID28 shows histologic features similar to that of PAC120. (**H**) At 3 days after docetaxel treatment, there is accumulation of mitotic cells, multipolar divisions and few apoptotic bodies (arrows). (**I**) At 3 days after treatment with the docetaxel plus trastuzumab combination, note the presence of enlarged and mitotic tumour cells in the middle of compact cellular masses, surrounded by elongated cells (arrows). Percent indicates the mitotic index.

PAC120 and HID28 are high-grade adenocarcinomas that display similar differentiation with rare glandular structures, whereas HID25 is mucinous as previously described (Legrier *et al*, 2004) (Figure 2A, D and G). Mitoses were numerous in all tumours (mitotic index was 21, 8 and 11% in PAC120, HID25 and HID28, respectively). At 3 days after docetaxel treatment, many cells were blocked in mitosis, increasing the mitotic index to 40, 29 and 56% in PAC120, HID25 and HID28, respectively. Nuclear abnormalities, such as multinucleation and multipolar division, were numerous (Figure 2B, E and H). At 3 days after the docetaxel plus trastuzumab combination treatment, the mitotic index was of 13, 5 and 20% in PAC120, HID25 and HID28, respectively (Figure 2C, F and I). Apoptosis indexes, evaluated by anti-caspase 3 antibody immunostaining, remained low in all xenografts, whether treated or not. In all tumours treated with the docetaxel plus trastuzumab combination, tumour cells appeared elongated with hyperchromatic nuclei appeared 3 days after treatment. This morphological alteration was not apparent after single-agent treatment (Figure 2I). After 47 days (three cycles of treatment), residual tumours were composed of small foci of cells within a stromal matrix (Figure 2C). Treatment with trastuzumab alone did not affect any histological parameter (data not shown).

### Treatment-induced molecular alterations in HID28 prostate cancer xenografts

**Figure 3:**
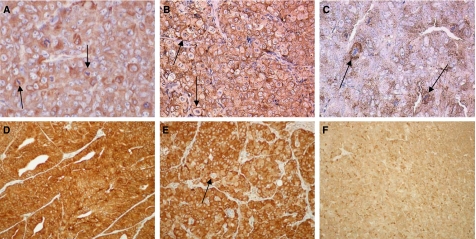
Expression pattern of HER2 and HSP90 in HID28 xenografts by immunohistochemistry. Left two panels, untreated tumours; middle two panels, docetaxel-treated tumours at day 3; right two panels, docetaxel/trastuzumab treatment (× 200 magnification). HER2 immunodetection. (**A**) The anti-HER2 antibody staining is weak, diffuse and focal, stronger in mitotic cells (arrows). (**B**) HER2 staining is increased in all the tumour cells with preferential membrane staining. (**C**) Immunostaining was restricted to the membrane of mitotic cells at day 15. HSP90 immunodetection. (**D**) Anti-HSP90 antibody stained the cytoplasm of HID28 tumour cells. (**E**) All tumour cells are stained except mitotic cells (arrow). (**F**) The anti-HSP90 staining disappeared from all tumour cells, apart from rare cells at day 1.

**Figure 4:**
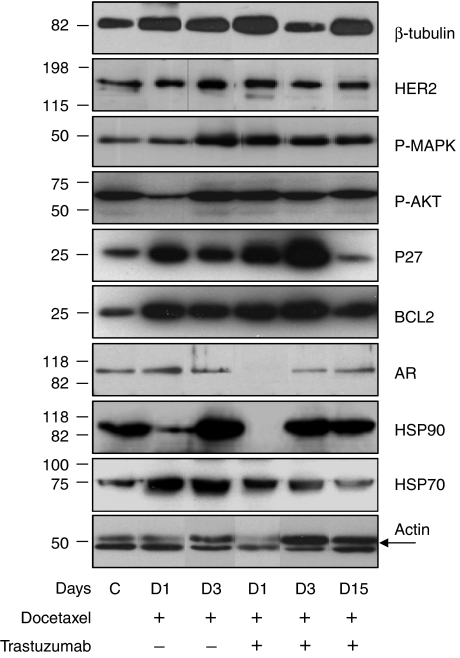
Western blotting analysis of protein expression in a representative HID28 xenograft, after one cycle of treatment. Untreated tumours (lane 1), 1 or 3 days after docetaxel treatment (lanes 2 and 3, respectively); 1, 3 or 15 days after docetaxel plus trastuzumab treatment (lanes 4, 5 and 6, respectively). Actin (45 kDa) was used as a control. Signals correspond to *β*-tubulin (110 kDa), HER2 (185 kDa), phospho-ERK (42–44 kDa), phospho-AKT (60 kDa), p27 (27 kDa), BCL2 (26 kDa) AR (110 kDa), HSP90 (90 kDa) and HSP70 (70kDa). Histograms of proteic expression of HER2, AR and HSP90 in treated tumours reported to that of controls, day 1, 3 or 15 after docetaxel treatment alone or combined with trastuzumab.

Western blotting (Figure 4) was used to evaluate changes in expression of *β*-tubulin, HER2, p-ERK, p-AKT, p27, BCL2, AR, HSP90 and HSP70 induced by treatment with docetaxel alone or the trastuzumab plus docetaxel combination in HID28 tumours, a very high responder. Tumour samples were harvested from groups of three mice and analysed 1, 3 and 15 days after one cycle of treatment. There were few variations between triplicate data corresponding to the three mice per group; consequently, the results shown corresponded to one representative mouse. The chosen kinetics allowed to evaluate optimal expression of different proteins of interest. Total *β*-tubulin increased after both treatment, except at day 3 after combination. HER2 protein levels (basal level at 0.47 in arbitrary units (a.u.) in controls, decreasing after 24 h) increased (two-fold) at day 3 after docetaxel treatment and at 24 h after the docetaxel plus trastuzumab combination then decreased at 3 and 15 days after combination treatment. At 3 days after docetaxel treatment, anti-HER2 antibody stained the membrane of tumour cells, more intensively than in the control group (Figure 3A and B). At day 15, in the group treated with the combination, HER2 expression was lower, restricted to the membrane of mitotic cells (Figure 3C). In the serum of control mice bearing HID28 xenografts, the median level of soluble HER2 was 1.1 ng ml^−1^. At 3 days after docetaxel treatment, it increased to more than 18 ng ml^−1^ (saturation of the kit) showing a release of free HER2 in the serum of three treated mice. Trastuzumab treatment alone did not induce at any time any change in the protein expression profile of HID28.

Phospho-ERK was strongly increased 3 days after docetaxel and 1, 3 and 15 days after the docetaxel/trastuzumab combination. Phospho-AKT (Ser473) decreased transiently 24 h after docetaxel treatment and returned to its initial level 3 days after treatment. Globally, no alteration of p-AKT level was observed except a decrease 24 h after the docetaxel/trastuzumab combination treatment. The amount of p27 CDK inhibitor protein was increased 1 and 3 days after both treatments and returned to control levels after 15 days. Elevation of BCL2 was immediate after both treatments and decreased 15 days after combination.

The androgen receptor was expressed at constant levels in tumours from untreated mice (basal level at 0.32 a.u.) or from mice treated with docetaxel alone. Androgen receptor (AR) expression was undetectable 24 h after the docetaxel plus trastuzumab treatment, coming back to low levels at day 3 with a complete restoration by day 15. HSP90 expression followed a similar pattern. After docetaxel treatment, anti-HSP90 antibody homogeneously stained the cytoplasm of tumour cells, except in mitotic cells (Figure 3D and E). After 24 h of combination treatment, the anti-HSP90 staining disappeared, consistent with Western blotting data (Figure 3F). HSP70 protein levels in Western blotting increased after docetaxel alone or combined, then progressively decreased.

**Figure 5:**
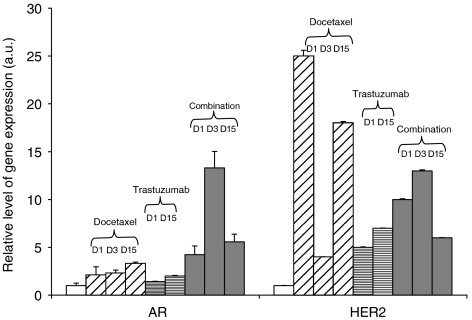
Real-time RT–PCR quantitation of AR and HER2 mRNA transcripts in HID28 xenograft, after one cycle of treatment. Median levels of five tumours for each treatment group. Gene expression, performed in triplicate, was normalised against the HPRT gene and expressed relative to the control group. Bars represent s.d.

The levels at HER2 and AR mRNA were analysed by real-time RT–PCR (Figure 5). Compared with control group (basal level 0.13 a.u), AR mRNA increased two-fold 24 h after docetaxel treatment and remained at this level after 3 and 15 days. After treatment with trastuzumab alone, AR mRNA levels were unchanged. The docetaxel plus trastuzumab combination induced a four-, 13- and five-fold increase in AR mRNA 1, 3 and 15 days after treatment, respectively. This contrasts with the total disappearance of AR protein day after the combination treatment and with AR protein levels still below baseline at day 3. The expression of HER2 mRNA was detected in control HID28 tumours (basal level at 0.01). Reverse transcriptase–polymerase chain reaction data showed an increased expression of HER2 mRNA after trastuzumab treatment less than after docetaxel alone or combination. HER2 mRNA levels were increased at all time points by all treatments, between 4–25-fold.

## DISCUSSION

The use of taxanes for treating hormone refractory prostate cancers has started to change the outcome of these patients; but, although encouraging, the therapeutic benefits need to be improved. To reach this goal, a better understanding of the biological processes initiated in taxane-treated tumours might lead to a design of more effective combination therapies. In a previous publication, we showed that the sensitivity of PAC120 prostate tumour and its HID variants xenografts to docetaxel (Oudard *et al*, 2003) was related with the constitutive level of HER2 mRNA expression. Here, we reported the potentiating effects of combining the docetaxel with trastuzumab, a humanised HER2 antagonistic antibody. Trastuzumab treatment alone was inactive in PAC120 xenografts and in HID28 variant, distinguishing our model from the CWR22 prostate cancer xenograft model, which overexpresses HER2 and is sensitive to Trastuzumab (Agus *et al*, 1999). This absence of response to trastuzumab alone has recently been reported in clinical trial in prostate cancer (Lara *et al*, 2004). Trastuzumab response in tumour types other than breast carcinoma remains to be determined. Docetaxel treament reduced tumour growth of the three tumour variants. Antitumoural effects induced by the docetaxel/trastuzumab combination were superior to that induced by docetaxel alone, in all growth parameters, percent of inhibition, survival prolongation and complete regressions. The effects of the combination docetaxel/trastuzumab have already been reported in breast cancer, but are not so clear. Few clinical trials have been conducted in prostate cancer (Small *et al*, 2001; Lara *et al*, 2004) with some encouraging data in the treatment of advanced prostate cancer.

Histology of docetaxel-treated tumours showed alterations typical of taxanes, such as cell cycle arrest and abnormal mitoses. Additionally, after docetaxel/trastuzumab combination treatment, numerous tumour cells appeared elongated, like ‘flattened balloons’ at the interface between tumour cords and murine stroma, whereas tumour cells are typically round in the not-treated tumours. This observation suggested a reduction of tumour cell tonicity, due to alterations of cytoplasmic, microtubules, induced by docetaxel treatment and potentiated by trastuzumab. We concluded that docetaxel had the expected effects on the mitotic spindle and the cytoskeleton and we postulated that trastuzumab enhanced the alterations of microtubules by inhibiting an HER2-dependent repair through a reduced phosphorylation. Our data pointed to a causal relationship between HER2 signalling, docetaxel-induced alterations of microtubules and potentiated tumour cell death by trastuzumab.

The primary target of taxanes is *β*-tubulin, which is part of the mitotic spindle and of cytoplasmic microtubules. Taxanes block tubulin depolymerisation, resulting in impairment of cellular mitosis (Horwitz, 1994), generating nuclear abnormalities and multipolar divisions, ultimately leading to cell death (Abal *et al*, 2003). Taxane-induced tubulin alterations may also affect microtubule-associated proteins (MAPS) (Burns *et al*, 1984, Haycock *et al*, 1992), potentially leading to multiple cellular dysfunctions. Microtubule-associated proteins are among the protein clients of heat-shock proteins (HSP70 and HSP90), themselves associated with and stabilising numerous proteins such as HER2, AKT and the AR (Georget *et al*, 2002). Numerous studies indicate that the function of MAPs in microtubule integrity is regulated by phosphorylation reactions via kinases (Horne and Guadagno, 2003).

Docetaxel alone or combined to trastuzumab affected the expression of several proteins involved in HER2 signalling in our xenograft model. Elucidation of differences of protein expression and interaction between different pathways will be important in the design of targeted treatment of advanced prostate cancer. We found that tumour HER2 mRNA increased 24 h after docetaxel treatment, followed at 72 h by an increase in HER2 protein and release of free HER2 in the serum of treated mice. Detection of HER2 at different times might correspond to restart in the cycle of survival cells. The role of HER2 in prostate cancer progression is still debated, with divergent reports about the level of HER2 expression in HRPC (Reese *et al*, 2001; Shi *et al*, 2001; Di Lorenzo *et al*, 2002; Fossa *et al*, 2002; Mendoza *et al*, 2002; Savinainen *et al*, 2002; Calvo *et al*, 2003; Hernes *et al*, 2004). These discrepancies could be due to the methodology used for detecting HER2, designed for detection in breast cancers, and it might be inadequate for prostate cancer tissue (Simon *et al*, 2001). In the present study, an overnight incubation with the antibody was performed. Alternatively, HER2 overexpression may play a role in hormonal escape in only a subset of tumours, since other potential mechanisms, such as increased AR expression or mutations in the AR, have also been described (Edwards *et al*, 2003). The increase in HER2 and phospho-ERK levels following docetaxel treatment is consistent with the notion that MAPKinase activation constitutes a cell survival/repair response to docetaxel. Similar conclusions were drawn from studies in which combinations of taxanes and pharmacological MAPK inhibitors were additive and correlated with MAPK activation following taxane treatment in various tumour cell lines (McDaid and Horwitz, 2001; Zelivianski *et al*, 2003).

Docetaxel alone reduced HSP90 protein level 24 h after treatment, but did not affect that of AR. Combined treatment induced a simultaneous disappearance of both AR and HSP90, indicating their close relationship. The loss of the AR protein occurred in spite of an increase in AR transcription, suggesting post-translational degradation. It is known that cytoplasmic AR degradation is prevented by its binding to heat-shock protein HSP90 (Georget *et al*, 2002). This could account for the potentiating effect of trastuzumab on docetaxel in the specific context of prostate cancer cells. Indeed, several recent studies suggest that AR continues to play a key role in the survival and proliferation of HID prostate cancers via HER2 signalisation (Craft *et al*, 1999; Yeh *et al*, 1999), explaining the role of trastuzumab in the combination with docetaxel.

However, in the present study, the potentiation of docetaxel by trastuzumab was not paralleled by a decrease in either phospho-ERK or phospho-AKT levels, suggesting that inhibition of another signalling pathway was involved in the degradation of AR/HSP90. In support of this possibility, a recent study using a dual EGFR/HER2 kinase inhibitor in prostate cancer cell lines showed that an HER2/ERBB3 signalling pathway protected the AR from degradation at low androgen concentrations through an AKT-independent pathway (Mellinghoff *et al*, 2004).

Our finding that docetaxel can induce an increase of HER2 transcription and expression in a human prostate cancer model is paralleled by a release of HER2; detection of seric HER2 could be searched for in patients after docetaxel treatment. Our data show that docetaxel/trastuzumab combination caused a simultaneous hormone-receptor and HSP90 degradation and enhances the antitumour efficacy of docetaxel, suggesting that complete inactivation of AR could affect HID prostate cancers. This provides preclinical support for exploring this therapeutic strategy in HRPC patients.

## Figures and Tables

**Table 1:** Effects of docetaxel alone or with trastuzumab on growth parameters of PAC120 and HID25 and HID28 variants

**Tumour**	**Treatment**	**Tumour GD in days^a^**	**Survival delay in days^b^**	**Tumour regression^c^**	**Cures^d^**
PAC120	None	20	33	0/9	0
	Docetaxel	36	64	1/9 (11%)	0
	Trastuzumab	20	33	0/8	0
	Docetaxel/trastuzumab	64	>64	7/8 (88%)	1
HID25	None	29	47	0/10	0
	Docetaxel	67	>92	0/11 (0%)	0
	Docetaxel/trastuzumab	>92	>92	11/13 (85%)	6 (46%)
HID28	None	15	35	0/10	0
	Docetaxel	82	100	4/10 (40%)	0
	Trastuzumab	14	32	0/8	0
	Docetaxel/trastuzumab	>132	>132	7/8 (88%)	5 (71%)

a Tumour growth delay (GD) was calculated as the median number of days required to observe a five times increase in initial tumour volume.

b Survival median delay in days after start of treatment.

c Number of mice with a reduction of tumour size after start of treatment to the total number of mice.

d Number of mice without tumour (no regrowth) at the end of experiment (killing of mice) out of the total number of mice.
